# Baseline NT-Pro-BNP Levels and Arrhythmia Recurrence in Outpatients Undergoing Elective Cardioversion of Persistent Atrial Fibrillation: A Survival Analysis

**Published:** 2009-01-07

**Authors:** Tommaso Sanna, Andrea Sonaglioni, Maurizio Pieroni, Antonio Dello Russo, Gemma Pelargonio, Michela Casella, Evelina Zichichi, Giuseppe La Torre, Maria Lucia Narducci, Fulvio Bellocci

**Affiliations:** 1 Institute of Cardiology, Catholic University of the Sacred Heart, Rome, Italy; 2 Institute of Hygiene, Catholic University of the Sacred Heart, Rome, Italy

**Keywords:** NT-Pro-BNP, Cardioversion, Persistent Atrial Fibrillation, Arrhythmia Recurrence

## Abstract

**Background:**

Atrial fibrillation (AF) is the most common arrhythmia encountered in clinical practice. Elective electrical cardioversion is often performed in patients with persistent AF to attempt sinus rhythm (SR) restoration. However, AF recurrences are frequent after successful cardioversion and several predictors have been identified.

**Aim of the study:**

The present study was designed to prospectively analyse the correlation between NT-pro-BNP levels and AF recurrence in consecutive patients referred for electrical cardioversion of persistent atrial fibrillation.

**Results:**

Forty consecutive patients referred for elective cardioversion of AF were enrolled in the study. Cardioversion restored sinus rhythm in 34/40 patients but 2 of them presented an early recurrence of AF before discharge. Patients were then followed for 6 months to assess AF recurrence. Cox regression analysis was performed using the parameters found predictive on univariate survival analysis (NT-pro-BNP quartiles, beta-blockers). The only independent predictor of AF recurrence on Cox-regression analysis was a level of NT-pro-BNP in the fourth quartile (HR 3.21 95%CI 1.26-8.14, p=0.014). On receiver operating curve (ROC) analysis, NT-pro-BNP levels above 1707 pg/ml had a specificity of 92% (and a sensitivity of 36%) in predicting atrial fibrillation recurrence by 6 months.

**Conclusions:**

Baseline NT-pro-BNP levels are an independent predictor of AF recurrence at 6 months follow-up in candidates for elective direct current cardioversion.

## Background

Atrial fibrillation (AF), with an estimated prevalence of 2.3 million patients in the US, is the most common arrhythmia encountered in clinical practice and represents the first cause of hospitalization for rhythm disturbances [1,2]. Elective electrical cardioversion represents an important therapeutic option in patients with persistent AF to attempt sinus rhythm (SR) restoration. However, AF recurrences are frequent after successful cardioversion and several predictors have been identified including AF duration [3-6], left atrial7,8 and left ventricular dimensions [4,5,9], left ventricular ejection fraction, echocardiographic characteristics,[10-12], electrocardiographic features[13,14], age[5,6,8], gender [15], body weight [16], underlying cardiac disease [4-6,8], NYHA functional class [5,19,20], previous relapses of AF [17,18], and their association [5].

Brain natriuretic peptide (BNP) is a molecule with vasodilative, diuretic and natriuretic activity, produced and secreted mainly by ventricular myocardium in response to stretching of cardiomyocytes [21,22] thus representing  a sensitive marker of left ventricular end-diastolic pressure. BNP is synthesized in the cardiomyocytes as an aminoacidic chain (pro-BNP) which is enzimatically cleaved into an inactive NH2- terminal fragment [23] (NT-proBNP) and the biologically active COOH-terminal fragment (BNP) [24]. Given its longer half-life (60-120 minutes vs. 22 minutes), NT-proBNP levels are more stable and less influenced by acute haemodynamic variations than BNP levels, and therefore reflect the mean values of left ventricular filling pressures over the previous 12 hours [25,26]. As left ventricular dysfunction and elevated filling pressure are associated with an increased probability of atrial fibrillation recurrence NT-pro-BNP, has been identified as a strong predictor of atrial fibrillation recurrence in different clinical settings. In particular, brain natriuretic peptide predicted AF after cardiac pacemaker implantation for sick sinus syndrome [27], postoperative AF in patients undergoing cardiac surgery [28], sinus rhythm maintenance 2 weeks after cardioversion [29] and AF recurrence in patients with NHYA functional classes I or II [30].

The present study was designed to prospectively analyse the correlation between NT-pro-BNP levels and AF recurrence in consecutive patients referred for electrical cardioversion of persistent atrial fibrillation, irrespective of NHYA functional class.

## Methods

### Patient population

Sixty-four consecutive patients referred to the outpatient clinic of the cardiovascular medicine department of our Institution for elective cardioversion of persistent AF between January 2006 and December 2006 were prospectively screened for inclusion in the study. Twenty-four patients were excluded from the study due to chronic renal failure, known to affect NT-pro-BNP levels. The remaining forty patients gave their informed consent and represent the study population. The study was approved by the institutional review board of our hospital.

### NT-pro-BNP 

A blood sample was drawn shortly before cardioversion with the patient at rest in supine position, to assess the concentration of the NT-proBNP at baseline. NT-proBNP was assessed by ECLIA method (Immunoassay in Electrochemyluminscence) and Elecsys 1010/2010 immunoanalyser.

### Cardioversion

Synchronized cardioversion was performed during deep sedation with intravenous propofol delivering a stepwise sequence of impedance compensating biphasic shocks as follows: first discharge 100J; in case of failure a second discharge was delivered at 150 J; in case of failure a third discharge was delivered a 200 J; in case of failure of a 200J biphasic shock, attempts were terminated and the electrical cardioversion classified as ineffective.

### Follow-up

After elective cardioversion, patients were followed monthly for 6 months or until a common stopping date. Additional follow-up visits were performed at patient's request in the case of symptom recurrence.

### End-point

The primary end-point was AF recurrence at scheduled or additional follow-up visits in patients discharged in sinus rhythm after elective cardioversion. The secondary end-point was acute efficacy of elective cardioversion, defined as discharge from the outpatient clinic in sinus rhythm after cardioversion.

### Statistical analysis

Normal distribution of data was verified with Kolmogorov-Smirnov test. Data showing a normal distribution are presented as mean ± SD. Non-Gaussian variables are presented as median [range]. Categorical data are presented as proportion of cases or percentages. Comparisons between pairs of Gaussian variables were performed with Student t-test. Comparisons between non-Gaussian variables were performed with Mann-Whitney or Wilcoxon signed-rank test, as appropriate. Comparisons of proportions between groups were performed with chi-square test or Fisher's exact test, as appropriate. Univariate survival analysis was performed with Kaplan-Meier method. Univariate survival analysis was performed exploring the effect of age (expressed as tertiles), gender, AF duration (expressed as tertiles), baseline NT-pro-BNP levels (expressed as quartiles) ejection fraction (categorized as normal vs. depressed if below 50%), atrial dimensions (expressed as tertiles), NYHA functional class (I and II vs III and IV) use of beta blockers, class Ic antiarrhythmic drugs and amiodarone as predictors of AF recurrence. The level of significance of the differences among survival curves was assessed by Log-rank and Breslow tests. Multivariate survival analysis was performed by Cox regression analysis on those variables significantly affecting AF recurrence at univariate survival analysis. A ROC analysis has been planned to identify possible cut-offs to predict AF recurrence at 6 months. Statistical analysis was performed with SPSS 13.0 software package.

## Results

Forty consecutive patients referred for elective cardioversion of AF were enrolled in the study and their baseline characteristics are summarized in Table 1. Patient population was represented by 23 males and 17 females aged 66.4±11.4 years, with an AF duration of 6.5 (2-26) months. NHYA functional class was I in 17, II in 14, III in 8 and IV in 1 patient. NT-pro-BNP levels before elective cardioversion were 1002 pg/ml (357-8027).

### Acute efficacy

Cardioversion restored sinus rhythm in 34/40 pts but 2 of them presented an early recurrence of AF before discharge. Age, gender, LV ejection fraction, LA dimensions, LV dimensions, use of beta-blockers, digoxin, furosemide, class IC antiarrhythmic drugs, amiodarone, calcium-channel blockers, AF duration, cardiovascular risk factors and baseline NT-pro-BNP levels were similar in patients with successful and unsuccessful cardioversion at univariate analysis (Table 2)

### Atrial fibrillation recurrence

The univariate influence of age (tertiles), gender, baseline NT-pro-BNP levels (quartiles), AF duration (tertiles), left ventricular ejection fraction (categorized as normal vs. depressed, if below 50%), atrial dimensions (tertiles), ventricular diameter (tertiles), NHYA functional class (I and II vs III and IV), use of beta blockers, class Ic antiarrhythmic drugs, amiodarone, digoxin, furosemide, calcium channel blockers as predictors of time to AF recurrence was assessed by survival analysis in patients discharged in sinus rhythm after elective cardioversion.

**Figure 1:**
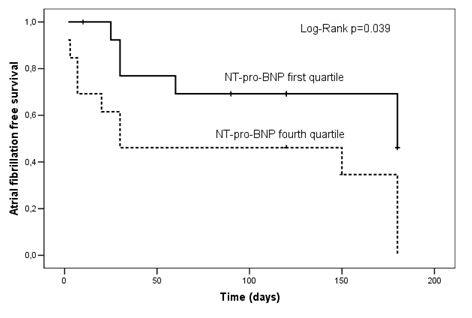
Kaplan-Meier curves of atrial fibrillation recurrence (first quartile compared to fourth quartile)

Kaplan-Meier survival curves were significantly different across quartiles of NT-pro-BNP levels (Log Rank Mantel-Cox  p=0.044; Breslow test p= 0.035; Taron-Ware p= 0.038). The results of the pre-specified comparisons between quartiles evidenced a higher recurrence rate in patients with NT-pro-BNP in the fourth quartile as compared to the first quartile (Log Rank Mantel-Cox  p=0.039; Breslow test p= 0.050; Taron-Ware p= 0.046). Kaplan-Meier survival curves (Figure 1) were also significantly different in pts treated with beta-blockers (Log Rank Mantel-Cox  p=0.037; Breslow test p= 0.053; Taron-Ware p= 0.043), with a higher recurrence rate in patients on treatment.

Cox regression analysis was performed using the parameters found predictive at univariate survival analysis (NT-pro-BNP quartiles, beta-blockers). The only independent predictor of AF recurrence at Cox-regression analysis was a level of NT-pro-BNP in the fourth quartile (HR 3.21 95%CI 1.26-8.14, p=0.014).

### Receiver operating curve analysis

**Figure 2:**
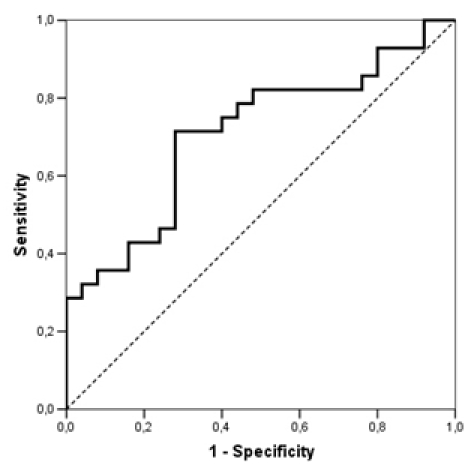
Receiver operating curve of NT-pro-BNP leveles as a predictor of AF recurrence

A ROC analysis of AF recurrence by 6 months as a function of baseline NT-pro-BNP levels has been performed. The area under the curve was 0.709 (95% C.I 0.568-0.849), p=0.009 (Figure 2). An analysis of NT-pro-BNP levels cut-offs was carried-out. NT-pro-BNP levels above 1707 pg/ml had a specificity of 92% (and a sensitivity of 36%) in predicting atrial fibrillation recurrence by 6 months.

## Discussion

Atrial fibrillation is the most common arrhythmia encountered in clinical practice. After a diagnosis of persistent AF, a rate control or a rhythm control strategy is alternatively pursued in individual patients, and excellent reviews have analysed the relative advantages and disadvantages. When a rhythm control strategy is considered, an accurate pre-procedural estimate of the probability of sinus rhythm maintenance after electrical cardioversion is likely to affect the decision. Several factors are known to be clearly predictive of poor results, as advanced age, enlarged atrium, depressed left ventricular ejection fraction, high NYHA functional class, and numerous previous recurrences.

Previous studies demonstrated that BNP levels, as a marker of left ventricular filling pressure, predict sinus rhythm maintainance 2 weeks after cardioversion [29] and that BNP plasma levels are an independent predictor of AF recurrence in different populations including patients with NHYA functional class I or II [30] submitted to electrical cardioversion.

However these findings appear to be of limited clinical value when extended to the whole spectrum of patients with persistent AF referred for possible cardioversion either because of the short term follow-up or because limited to patient in NHYA functional class I or II.

Our study confirms previous findings and demonstrate that high NT-proBNP levels at baseline in candidates for elective cardioversion are independently predictive of AF recurrence at 6 months follow-up irrespectively of NYHA functional class.

These findings suggest that assessment of BNP levels in candidates to electrical cardioversion may represent an important information to decide whether and when to perform attempts to restore sinus rhythm. In fact, as BNP levels mostly reflect left ventricular filling pressure and then atrial pressure and stretch, it can be suggested that therapy with drugs affecting filling pressure, such as diuretics and ACE-inhibitors or angiotensin-receptor blockers, could be modulated in order to reduce BNP levels thus increasing the success rate of cardioversion and sinus rhythm maintenance. Accordingly it is well established that pre-treatment with angiotensin-receptor blockers increase the probability of sinus rhythm maintenance in patients with persistent atrial fibrillation subjected to cardioversion [31]. Interestingly in our study ejection fraction was not predictive of AF recurrence: although we cannot exclude that the study sample was not large enough to demonstrate the well established role of ejection fraction, our findings suggest that the increase of left ventricular filling pressure, not necessarily reflecting a worse contractile function, represents the key player in determining atrial fibrillation occurrence and recurrence in patients with cardiac dysfunction.

Our findings have relevant clinical implications as they identify NT-pro-BNP levels as an important tool to estimate the probability of AF recurrence in the population of patients referred for electrical cardioversion, including patients with variable degrees of cardiac dysfunction and different NHYA functional classes. A low probability of sinus rhythm maintenance, as estimated on the basis of patients characteristics, including baseline NT-pro-BNP levels, might suggest the physician to prefer a rhythm control to a rate control strategy and possibly to adopt therapeutic strategies aimed to reduce left ventricular filling pressure, and therefore BNP levels, to increase the rate of success of cardioversion. Cut off levels suggesting inefficacy of cardioversion attempts have been proposed, which need to be confirmed by further investigations.

## Study Limitations

We want to recognize several limitations of our study. Recurrence of AF was detected at visits elicited by return of symptoms or at scheduled monthly visits, underestimating AF recurrences and giving an inaccurate estimation of time to recurrence. Even though no restrictions were set on NHYA class, only 28% of patients discharged in sinus rhythm were in NHYA functional III or IV resulting in a limited contribution to our case-mix.

## Conclusions

Baseline NT-proBNP levels are an independent predictor of AF recurrence at 6 months follow-up in candidates for elective direct current cardioversion.

## Figures and Tables

**Table 1:**
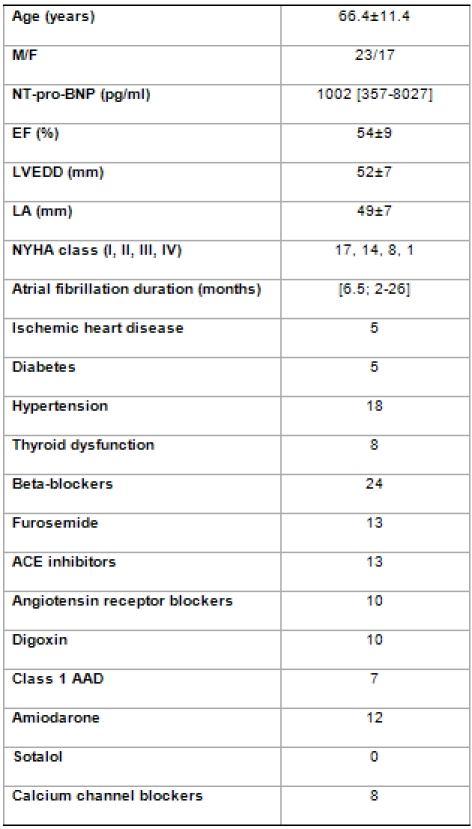
Baseline characteristics

EF: left ventricular ejection fraction; LVEDD: left ventricular end-diastolic diameter; LA: left atrial diameter.

**Table 2:**
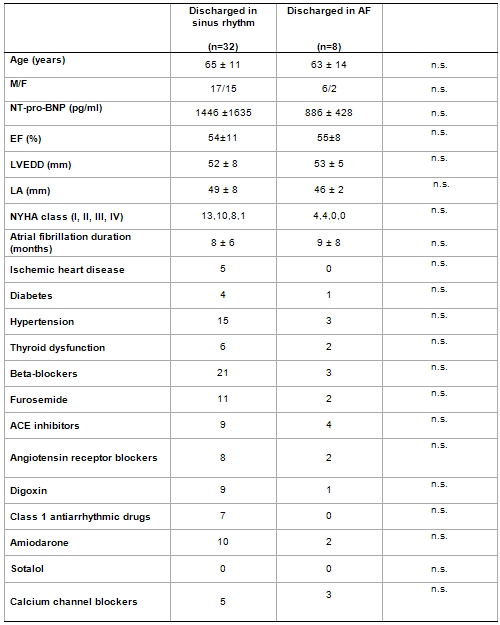
Comparison of baseline characteristics in patients discharged in sinus rhythm compared to pts discharged in atrial fibrillation

EF: left ventricular ejection fraction; LVEDD: left ventricular end-diastolic diameter; LA: left atrial diameter.
